# Overcharging Effect in Electrospray Ionization Mass Spectra of Daunomycin-Tuftsin Bioconjugates

**DOI:** 10.3390/molecules24162981

**Published:** 2019-08-16

**Authors:** Lilla Pethő, Gábor Mező, Gitta Schlosser

**Affiliations:** 1 MTA-ELTE Research Group of Peptide Chemistry, Hungarian Academy of Sciences, Eötvös Loránd University, Pázmány Péter sétány 1/A, 1117 Budapest, Hungary; 2 Department of Organic Chemistry, Institute of Chemistry, Faculty of Science, ELTE Eötvös Loránd University, Pázmány Péter sétány 1/A, 1117 Budapest, Hungary; 3 Department of Analytical Chemistry, Institute of Chemistry, Faculty of Science, ELTE Eötvös Loránd University, Pázmány Péter sétány 1/A, 1117 Budapest, Hungary

**Keywords:** bioconjugates, peptides, anthracyclines, mass spectrometry, electrospray ionization, fragmentation

## Abstract

Peptide-based small molecule drug conjugates for targeted tumor therapy are currently in the focus of intensive research. Anthracyclines, like daunomycin, are commonly used anticancer drug molecules and are also often applied in peptide-drug conjugates. However, lability of the *O*-glycosidic bond during electrospray ionization mass spectrometric analysis hinders the analytical characterization of the constructs. “Overprotonation” can occur if daunomycin is linked to positively charged peptide carriers, like tuftsin derivatives. In these molecules, the high number of positive charges enhances the in-source fragmentation significantly, leading to complex mass spectra composed of mainly fragment ions. Therefore, we investigated different novel tuftsin-daunomycin conjugates to find an appropriate condition for mass spectrometric detection. Our results showed that shifting the charge states to lower charges helped to keep ions intact. In this way, a clear spectrum could be obtained containing intact protonated molecules only. Shifting of the protonation states to lower charges could be achieved with the use of appropriate neutral volatile buffers and with tuning the ion source parameters.

## 1. Introduction

Daunomycin is a commonly used drug for cancer therapy, which is applied, especially in leukemia [1]. It belongs to the family of anthracycline antibiotics that can bind to the DNA in the nucleus and can inhibit the topoisomerase IIα enzyme [1,2]. The molecule consists of a tetracyclic quinoid aglycone part and an aminoglycoside sugar moiety (daunosamine), attached through an *O*-glycosidic bond to the aglycone part. Unfortunately, the use of daunomycin in clinical treatments is limited by severe side-effects, for example, cardiotoxicity. To overcome clinical drawbacks, such as the lack of selectivity, the fast elimination from the blood circulation, as well as toxicity, peptide-based bioconjugates containing daunomycin for targeted therapy have been in the focus of intensive interest since years [3,4,5]. The drug moiety, usually, is attached to a targeting peptide, which can bind specifically to a receptor overexpressed in tumor cells, and the selective binding results in a specific antitumor effect [6]. 

Tuftsin is a naturally occurring tetrapeptide (TKPR), a proteolytic fragment (289–292) of the immunoglobulin G (IgG) Fc heavy chain [7]. Tuftsin derivatives have already been applied in drug delivery systems using methotrexate as drug molecule [8,9]. Furthermore, the ε-amino group of the lysine provides a potential coupling site for the drug conjugation, and this branched structure can increase the stability of the bioconjugates in biological systems, too [10,11]. 

Chemical synthesis of multicomponent bioconjugates is usually based on a complex chemical strategy, in which mass spectrometry is a key technique for the fast and reliable identification of the products. However, in the case of anthracyclines and anthracycline derivatives, it is difficult to estimate the purity of the compounds due to the unusual fragmentation during MS analysis. 

**Figure 1:**
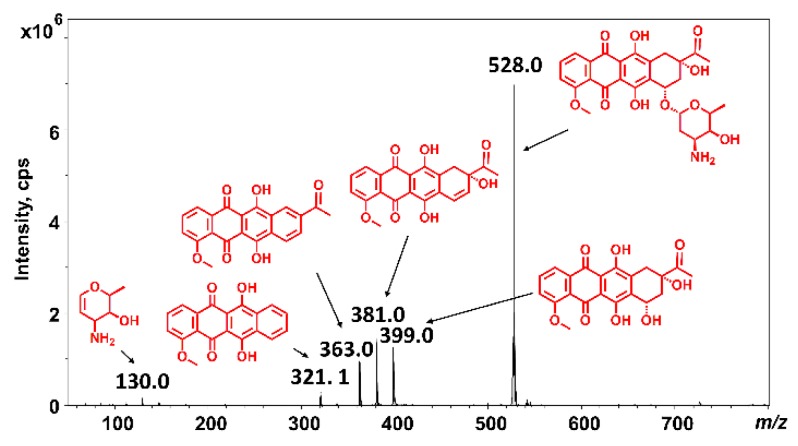
In-source fragmentation pattern of daunomycin (*m/z* 528.0, [M + H]^+^) under the commonly used electrospray ionization-mass spectrometry (ESI-MS) conditions. The structures correspond to the non-protonated molecules, and the protonation sites are not specified here.

Electrospray ionization (ESI) and matrix-assisted laser desorption ionization (MALDI) are soft ionization techniques, which usually produce intact protonated ions from peptides and proteins. However, mass spectra of daunomycin containing bioconjugates show significant fragmentation under the commonly used mass spectrometric conditions. Daunomycin itself usually produces a characteristic in-source fragment ion pattern (Figure 1). The main fragmentation pathway is the cleavage of the glycosidic bond, with the charge either residing on the aglycone or the daunosamine part [12]; therefore, the sugar moiety can be cleaved (*m/z* 130.0), resulting in hydroxyl functionality on the aglycone part (*m/z* 528.0 − 129.0 = 399.0) or eventuating in an unsaturated aglycone (*m/z* 528.0 − 147.0 = 381.0). Recently, an emerging number of research papers have been reported about the synthesis of novel anthracycline-containing bioconjugates [13,14,15,16,17]. However, in most cases, analytical data report complex mass spectra showing a mixture of protonated molecules, adduct ions, and various fragment ions, and publications lack proper assignation and discussion of the detected peaks. Therefore, homogeneity and structure of the compounds cannot be evaluated. We aimed to get an insight into the impact of structural and instrumental features affecting spectrum quality and to provide reliable analytical methodologies for the characterization of bioconjugates.

Daunomycin can be linked to peptides either via the sugar moiety with an amide bond or via the aglycon part with an oxime or hydrazone linkage. In the first case, the aglycon part [13], while in the latter case, the sugar moiety [14,15,16,17] can be cleaved from the Dau-containing molecule during the mass spectrometric analysis by in-source fragmentation, resulting in the above-mentioned complex spectra. However, the glycosidic bond can be cleaved under acidic conditions during the synthesis of the conjugate as well, and the loss of the daunosamine moiety leads to significantly decreased biological effects [18]. Therefore, the use of reliable mass spectrometric techniques with significantly suppressed in-source fragmentation processes is essential for the structural characterization of bioconjugates and the differentiation of synthetic by-products from the planned molecule. This could also facilitate the identification of other structural changes in the conjugates. A typical example is the *O* (aglycone) → *N* (daunosamine) acyl transfer of doxorubicin-peptide conjugates with ester linkage, where the different structures bear different biological effects while their molecular weight is the same. Therefore, it is highly important to characterize the structure properly and clarify whether the drug molecule is conjugated via an ester or an amide bond since the latter one is ineffective [19].

**Figure 2:**
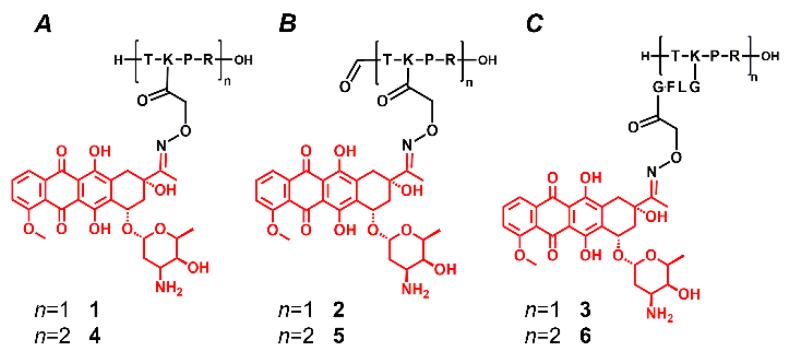
Schematic structure of the novel daunomycin-tuftsin bioconjugates.

Our research was focused on the detection of daunomycin-containing peptide conjugates and the determination of appropriate circumstances for the mass spectrometric characterization of these complex molecules. For this purpose, new tuftsin-based bioconjugates were synthesized to investigate the gas-phase stability of Dau in the presence of positively charged amino acid residues (Figure 2). The influence of structural elements on the fragmentation was studied in detail, including the (i) number of drug molecules; (ii) number of basic functional groups; (iii) presence or absence of a widely used enzyme-labile spacer (GFLG) between the targeting peptide and the drug molecule. We expected that these structural modifications, i.e., the reduction of the number of charged functional groups and the increased distance between the Dau and the peptide, would change the gas-phase stability of daunomycin and the fragmentation could be different. Besides, our main goal was to suppress the in-source sugar losses, and thereby to detect intact protonated molecules only. Therefore, we aimed to optimize the mass spectrometric conditions (ion source parameters and solvents), as well.

## 2. Results

### 2.1. Synthesis of the Conjugates

All peptides were synthesized by solid-phase methodology using the Fmoc/*t*Bu strategy. Boc-protected aminooxyacetic acid (Boc-Aoa-OH) was coupled directly or through a short enzyme labile spacer (GFLG) to the ε-amino group, that can be cleaved by the lysosomal enzyme cathepsin B, resulting in different metabolite (Dau=Aoa-Gly-OH) in comparison with a direct attachment that can serve H-Lys(Dau=Aoa)-OH metabolite after enzymatic decomposition. Dau was conjugated to the purified aminooxyacetylated peptides by oxime linkage under slightly acidic conditions (pH 5.1). The formed tuftsin-daunomycin bioconjugates and their dimer derivatives (containing two tuftsin units and hereby two aminooxy groups and two Dau) were purified by reversed-phase high-performance liquid chromatography (RP-HPLC). The purity of the products was analyzed by analytical HPLC (analytical data, chromatograms in Supplementary Materials: Table S1 and Figures S1–S14).

### 2.2. Mass Spectrometric Analysis under the Commonly Used Conditions

**Figure 3:**
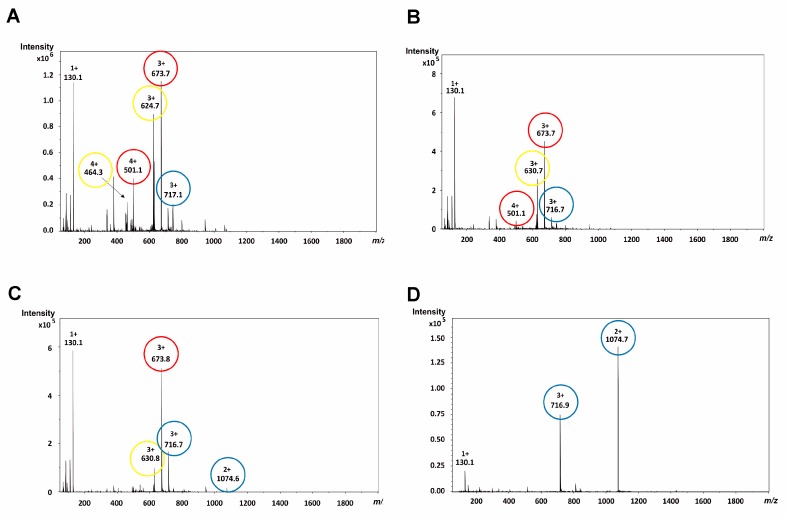
ESI-MS spectra of conjugate **4** (**A**) under the commonly used ion source parameters (136 V capillary exit potential); (**B**) using reduced capillary exit potential (5 V); (**C**) in non-acidic solvent mixture (acetonitrile-water 50:50% *v*/*v*; 5 V capillary exit potential); (**D**) measured in 50 mM NH_4_OAc buffer (pH = 6.7, diluted with acetonitrile 50:50% *v*/*v*; 5 V capillary exit potential). Blue circles are used to label intact protonated molecules, yellow and red circles label protonated fragment ions with one and two sugar losses, respectively. Charge states and *m/z* values of the protonated molecules are shown in the Figure.

The purified daunomycin-tuftsin conjugates were analyzed by ESI-MS (under the commonly used conditions, from an acetonitrile/water solvent mixture containing 0.1% acetic acid or formic acid), but the spectra showed several peaks. Among them, numerous peaks could be identified as fragment ions and only less than 40% of the peaks as intact protonated ions. Especially bioconjugates containing two daunomycin molecules (**4**, **5**, **6**) attached to the core peptide produced highly complex mass spectra. These bioconjugates contained two glycosidic bonds that could split independently during the mass spectrometric analysis, and the evaluation of the ESI-MS spectra was even more difficult due to the combination of the sugar losses (Figure 3A). Hence, the overcharging of daunomycin-tuftsin conjugates should be suppressed for unambiguous detection of the molecules and reliable analytical characterization of the products. 

According to our results, a free *N*-terminal amino group facilitated the cleavage of the glycosidic bond, while blocking the *N*-terminal by formylation (**2**, **5**), resulting in lower fragmentation, especially in the case of the monomer conjugate (**2**; Figure S3). Presence of the *N*-terminal formyl group was not enough to eliminate the in-source split of the sugar moiety, but it could decrease this phenomenon under the commonly used conditions. We also observed that the incorporation of a neutral spacer (GFLG) between the peptide and the drug moiety (**3**, **6**), hereby moving the sugar moiety away from the peptide backbone and the functional group with basic character, could not only increase the biological efficacy of the conjugates but also slightly decrease the in-source fragmentation during ESI-MS (**3**; Figure S5, and **6**; Figure S13). In the case of the monomer conjugate (**1**), a decrease of the fragmentation due to the blocking of the *N*-terminal amino group (**2**) or due to the introduction of the spacer moiety (**3**) resulted in simpler mass spectra with intact protonated molecules as base peaks.

### 2.3. Mass Spectrometric Analysis under the Changed Conditions

Our main goal was to achieve the detection of intact protonated ions; therefore, the optimization of the experimental conditions was performed to avoid overcharging and hereby inhibit the fragmentation of the glycosidic bond. In our experiments, we focused on the suppression of the in-source fragmentation phenomenon and investigated the effect of the capillary exit potential and the composition of the solvents used for the ionization of the samples.

The results showed that a significant reduction of the capillary exit potential could slightly increase the intensity of the intact protonated molecules. Use of the default capillary exit potential values always resulted in high fragmentation levels implying fragment ions ([MW-1 sugar] and/or [MW-2 sugars]) as base peaks in the mass spectra (Figure 3A). However, fragment ions were still dominant for conjugates containing a free *N*-terminal amino group (**1**, **3**, **4**, **6**) using low capillary exit potential (5 V; Figure 3B), while formylation of the *N*-terminal (**2**, **5**) could significantly decrease the number of sugar losses, especially the simultaneous loss of two sugar moieties. It is important to note that other ion source parameters did not affect the ion ratios significantly in the mass spectra.

Furthermore, we investigated the effect of using acidic vs. non-acidic solvent mixtures for ESI ionization while keeping the reduced capillary exit potential. We used acetonitrile-water (50:50%, *v*/*v*) mixture and solutions containing ammonium bicarbonate (NH_4_HCO_3_, 50 mM, pH 7.8) or ammonium acetate buffers (NH_4_OAc, 50 mM, pH 6.7) and acetonitrile (50:50%, *v*/*v*). Our results showed that the decrease in the number of charges on the protonated molecules formed during ESI ionization reduced the spontaneous dissociation of the glycosidic bond (Figure 3D). 

The non-acidic acetonitrile-water (50:50%, *v*/*v*) mixture could significantly decrease the amount of the fragment ions in case of conjugates **1**, **2**, **3**, and **5**, while the elimination of the acid was not effective for compounds **4** and **6**, though the simultaneous loss of two sugar moieties was suppressed (Figure 3C).

In case of the volatile buffers, intact protonated molecules of all bioconjugates were dominant. NH_4_HCO_3_ buffer (50 mM, pH 7.8) was less effective, noisy spectra with lower intensities and only ~80% intact protonated ions were detected, while NH_4_OAc buffer (50 mM, pH 6.7) provided clearer spectra and over 95% intact protonated ions.

Our results showed that the best quality spectra could be achieved with a combination of low capillary exit potential (5 V) and the charge-reducing NH_4_OAc buffer (50 mM, pH 6.7). In this case, only intact protonated molecules were found in the ESI-MS spectra of the bioconjugates. 

## 3. Discussion

There is an increased interest in peptide-based small molecule drug conjugates (SMDCs) for targeted tumor therapy. The most commonly used drugs in these constructs are anthracyclines; however, their lability during mass spectrometric analysis leads to sugar loss and makes it difficult to estimate the purity of the conjugates. The sugar loss is structure-dependent and can occur, especially in the case of the most common positively charged peptide carriers like tuftsin derivatives or many cell-penetrating peptides. Therefore, six new tuftsin-based daunomycin conjugates were prepared where Dau was linked to the aminooxyacetyl modified peptides via oxime linkage (Figure 2). Conjugates **1**, **2**, and **3** contained one tuftsin and one Dau molecule, while conjugates **4**, **5**, and **6** were their linear dimer derivatives with two tuftsin units and two Dau (Figure 2). Using *N*-terminal formylation (conjugates **2** and **5**; Figure 2B), the number of free amino groups was reduced. Furthermore, the incorporation of a peptide spacer (GFLG) between the targeting peptide and the drug molecule (conjugates **3** and **6**; Figure 2C) increased the distance between the positively charged amino acids and the daunomycin. This cathepsin B labile spacer can be cleaved in the lysosomes and increase the efficacy of the drug release [20], while it can change the analytical features, too.

In the ESI-MS spectra of the daunomycin-tuftsin conjugates, intact protonated ions are present in very low intensities only (<40%), which is an unusual feature in the ESI spectra of peptides, and it is a strong bottleneck of further mass spectrometry-based structural or analytical studies. Unfortunately, chemical degradation may also occur during the synthesis or the storage of these conjugates [13]. We also observed the cleavage of the sugar moieties in some conjugates after the RP-HPLC purification. This side reaction led to biologically less active by-products while having identical *m/z* values to the fragment ions (Figures S5 and S7); therefore, these species could not be differentiated under the commonly used MS conditions, and the purity of the compound could not be verified. Therefore, the development of appropriate conditions for efficient MS analysis is necessary.

We observed that the high number of charges on the peptide moiety induced a spontaneous dissociation of the glycosidic bond because of the repulsion of the positive charges. This phenomenon can be explained with the solvated proton theory. After ionization, H^+^ ions are localized on the most basic sites of the molecule, e.g., on the *N*-terminal and the side chain of basic amino acid (arginine, lysine, and histidine) residues. The population of different protonated forms depends on the internal energy content of the peptide and the gas-phase basicities of the different protonation sites. If an amino acid side chain (e.g., arginine) tightly binds a proton, then it can be solvated by other heteroatoms in the system. Location of the charge and the intramolecular hydrogen bonds can directly influence the structure and stability of the gas-phase ion [21,22]. The number and location of charges determine the extent of stability of the formed ions, and the too-high charge-repulsion can cause spontaneous fragmentation. Interesting examples are oligotuftsin peptides (repeating Thr-Lys-Pro-Arg peptide units [7]) since these peptides bear numerous protonation sites and produce ions with particularly high charge states using ESI-MS, hereby fragmentation can already occur in a single stage MS experiment due to charge repulsion. This phenomenon is defined here as “overcharging” or “overprotonation” of the analyte. We apply this term for cases, in which the high number of charges (protons) cause difficulties in a mass spectrometric experiment, especially due to the spontaneous dissociation of the analyte. From a practical point of view, overcharging is the opposite of supercharging. Supercharging is used for techniques, in which the number of charges is increased artificially. It is usually achieved by adding a supercharging reagent to the electrospray solution that increases the average charge of peptides and proteins. This facilitates high-resolution MS by reducing mass-to-charge (*m/z*) ratios and, therefore, improves peptide and protein identification in proteomics applications [23,24,25,26,27,28]. In the case of overcharging, the number of charges needs to be decreased artificially to obtain an ESI-MS spectrum with appropriate quality. Numerous methods for reducing the number of charges has been developed in the last few years. Ion/ion gas-phase reactions [29], ^210^Po α-particle source-based charge reduction [30,31], and interface acid vapor leak-in [32] have already been used successfully for multiply-charged proteins. Charge reduction can also be achieved by using basic buffer salts, such as ammonium acetate, or solution additives with strong gas-phase basicities that can decrease the acidification during ionization [33].

**Figure 4:**
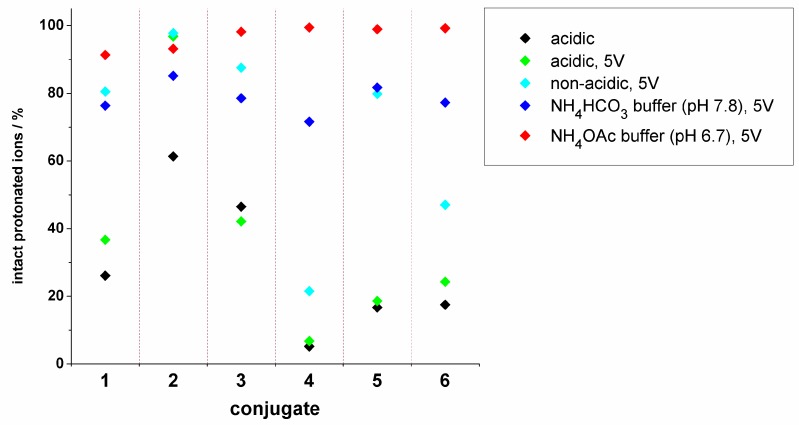
The intensity of the intact protonated ions (%) in the case of novel tuftsin-daunomycin conjugates under various conditions. The capillary exit potential, the solvents, and the pH were modified systematically.

The novel daunomycin-tuftsin conjugates behaved as expected under the commonly used conditions, i.e., a higher number of positively charged functional groups in the molecule indicated higher fragmentation. Consequently, intact protonated ions were detected in lower amounts in the case of the free *N*-terminal containing conjugates, while formylation of the *N*-terminal (**2** and **5**) resulted in significantly lower fragmentation. We also found that enhanced distance between the sugar moiety and the positively charged peptide backbone (incorporation of the GFLG spacer) decreased the fragmentation. According to these observations, we can conclude that special structural modifications can influence (enhance or decrease) the gas-phase stability of daunomycin, i.e., affect the split of the sugar moiety, but these effects are rather small. An overview of these data is represented in Figure 4.

We hypothesized that optimized ion source parameters or appropriate solvent mixtures used for the ionization would reduce the in-source fragmentation of the samples. Hence, we changed the capillary exit potential, and we could show that the reduction of this mass spectrometric parameter could slightly decrease the fragmentation. In the case of compound **2**, this modification in the mass spectrometric conditions resulted in a significantly improved spectrum, in which intact protonated ions were detected as base peaks (97%, Figure 4). The spontaneous dissociation of the glycosidic bond was facilitated by the highly charged peptide chain, therefore, shifting the charge states to lower charges could help to keep ions intact during ESI-MS analysis. Hence, application of neutral or slightly basic volatile buffers for the ESI-MS measurements could significantly reduce the fragmentation of the analyte and, therefore, reduce the amount of the sugar lost ions in the spectra. In our experiments, the most appropriate buffer for suppressed fragmentation was ammonium acetate (pH 6.7). NH_4_OAc buffer in acetonitrile combined with low capillary exit potential (5 V) could significantly reduce the charge states of various daunomycin-tuftsin conjugates and decrease their in-source fragmentation (Figure 4). 

In conclusion, not only the settings of the mass spectrometer but also the structure of the daunomycin-tuftsin conjugates had a high impact on the ESI-MS spectra. Though structural changes can assist the reduction of the fragmentation, overprotonation can only be suppressed with appropriate solvents and optimized mass spectrometric settings. These conditions can be useful in the analysis of anthracycline-containing bioconjugates in general, to obtain mass spectra comprising intact protonated molecules only.

## 4. Materials and Methods 

### 4.1. Chemicals

All amino acid derivatives, the Wang resin, *N*,*N*’-diisopropylcarbodiimide (DIC), and trifluoroacetic acid (TFA) were purchased from Iris Biotech GmbH (Marktredwitz, Germany). Boc-aminooxyacetic acid (Boc-Aoa-OH), 1-hydroxybenzotriazole hydrate (HOBt), 4-(dimethylamino)pyridin (DMAP), triisopropylsilane (TIS), and hydrazine hydrate were obtained from Sigma Aldrich Kft. (Budapest, Hungary). Aminooxyacetic acid, 1,8-diazabicyclo[5.4.0]undec-7-ene (DBU) were from TCI Europe N.V. (Zwijndrecht, Belgium), while piperidine was purchased from Molar Chemicals Kft (Budapest, Hungary). Formic acid 2,4,6-trichlorophenyl ester (For-OTcp) was prepared in our laboratory from formic acid and 2,4,6-trichlorophenol with *N,N′*-dicyclohexylcarbodiimide (DCC) (Sigma Aldrich Kft. Budapest, Hungary). Daunomycin hydrochloride was a kind gift from IVAX (Budapest, Hungary). All solvents used for synthesis and purification were purchased from VWR International Kft. (Debrecen, Hungary). 

Solvents and salts (NH_4_OAc, NH_4_HCO_3_) for the mass spectrometric measurements were purchased from Sigma Aldrich Kft. (Budapest, Hungary). Double distilled water was used for sample preparation.

All reagents and solvents were of analytical grade or highest available purity. 

### 4.2. Preparation of Daunomycin-Peptide Bioconjugates

The peptides were synthesized manually by solid-phase peptide synthesis using the Fmoc/*t*Bu strategy on Wang resin (0.6 mmol/g capacity) in a funnel equipped with a filter. The first amino acid (Fmoc-Arg(Pbf)-OH, 2 equiv. to the resin capacity) was coupled using an equivalent amount of DIC in *N*,*N*-dimethylformamide (DMF) in the presence of 0.1 equiv. DMAP to the amino acid derivative. All other amino acids were coupled with DIC/HOBt (3 equiv. each to the resin capacity) in DMF. Fmoc group was cleaved with 2% piperidine + 2% DBU in DMF in four steps (2+2+5+10 min). Peptides with free *N*-terminus were synthesized using Boc-Thr(*t*Bu)-OH as the last amino acid of the peptide backbone. The *N*-terminal of formylated derivatives was reacted with 2,4,6-trichlorophenyl formate. Then, the lysine side chain protecting group (Dde) was selectively cleaved with 2% hydrazine hydrate in DMF on resin, and Boc-protected aminooxyacetic acid was coupled either directly to the ε-amino group of the lysine or to a GFLG spacer that was built up earlier on the lysine side chain with the standard Fmoc/*t*Bu protocol. Peptides were cleaved from the resin (and all protecting groups were also removed) with 95% trifluoroacetic acid, 2.5% water, 2.5% TIS (*v*/*v*/*v*) in the presence of 10 equiv. free aminooxyacetic acid as “carbonyl capture” reagent [16] for 30 min at 0 °C, then 2 h at room temperature followed by precipitation with ice-cold diethyl ether, washing three times with diethyl ether and solubilization in distilled water for freeze-drying. The crude peptides were purified by RP-HPLC, and the pure fractions were immediately used for the next synthetic step after evaporation of the solvent. Daunomycin was conjugated to the peptides in solution (0.2 M NH_4_OAc, pH 5.1) at a peptide concentration of 10 mg/mL. The reaction mixtures were stirred at room temperature for 16 h, and the resulting bioconjugates were purified by RP-HPLC. The purity of the conjugates was investigated by analytical HPLC using a Knauer HPLC system and a Nucleosil C18 column (5 μm, 100 Å; 250 × 4.6 mm). Eluents were 0.1% TFA in water (A) and 0.1% TFA in acetonitrile-water 80:20%, *v/v* (B). All chromatographic separations were performed at room temperature.

### 4.3. Mass Spectrometry

Mass spectrometric experiments were performed by electrospray ionization on a Bruker Daltonics Esquire 3000+ (Bruker Daltonic GmbH, Bremen, Germany) ion trap mass spectrometer, operating with continuous sample injection at 10 μL/min flow rate. Mass spectra were recorded in positive ion mode in the *m/z* 50–2000 range, with 250 °C heating and N_2_ as nebulizer (10 psi) and dry gas (4 L/min). Stock solutions were prepared in double-distilled water (*c_stock_* = 1 nmol/µL) and diluted with the solvent mixtures. Samples were analyzed in acetonitrile-water (50:50%, *v/v*) mixture with or without 0.1% acetic acid (*c_conj.;acidic_* = 10 pmol/µL and *c_conj.;non-acidic_* = 20 pmol/µL) and solutions containing NH_4_HCO_3_ (pH 7.8) or NH_4_OAc buffers (pH 6.7) and acetonitrile (50:50%, *v/v*, final buffer concentration was 50 mM; *c_conj.;buffer_* = 50 pmol/µL). 

## Supplementary Materials

The following are available online, Table S1: Characteristics of daunomycin-tuftsin conjugates, Figure S1: ESI-MS spectra of **1**, Figure S2: Analytical HPLC chromatogram of **1**, Figure S3: ESI-MS spectra of **2**, Figure S4: Analytical HPLC chromatogram of **2**, Figure S5: ESI-MS spectra of **3**, Figure S6: Analytical HPLC chromatogram of **3**, Figure S7: ESI-MS spectrum of sugar-lost, purified **3**, Figure S8: Analytical HPLC chromatogram of sugar-lost **3**, Figure S9: ESI-MS spectra of **4**, Figure S10: Analytical HPLC chromatogram of **4**, Figure S11: ESI-MS spectra of **5**, Figure S12: Analytical HPLC chromatogram of **5**, Figure S13: ESI-MS spectra of **6**, Figure S14: Analytical HPLC chromatogram of **6**.
