# Supplementary material for: Overcharging Effect in Electrospray Ionization Mass Spectra of Daunomycin-Tuftsin Bioconjugates
Source: Molecules. 2019 Aug 16;24(16):2981. doi: 10.3390/molecules24162981 (PMC6720970; doi:10.3390/molecules24162981)
Supplement: Supplementary file 1 [file molecules-24-02981-s001.pdf]

Supplementary Materials

Overcharging effect in electrospray ionization mass spectra of daunomycin-tuftsin bioconjugates

Lilla Pethő [1], Gábor Mező [1,2] and Gitta Schlosser [1,3,*]

[1] MTA-ELTE Research Group of Peptide Chemistry, Hungarian Academy of Sciences, Eötvös Loránd University, Pázmány Péter sétány 1/A, 1117 Budapest, Hungary
[2] ELTE Eötvös Loránd University, Faculty of Science, Institute of Chemistry, Department of Organic Chemistry, Pázmány Péter sétány 1/A, 1117 Budapest, Hungary
[3] ELTE Eötvös Loránd University, Faculty of Science, Institute of Chemistry, Department of Analytical Chemistry, Pázmány Péter sétány 1/A, 1117 Budapest, Hungary
* Correspondence: schlosser@caesar.elte.hu

TABLE OF CONTENT

Analytical data......S2
Summarized analytical data of the daunomycin-tuftsin bioconjugates......S2
Mass spectra and analytical RP-HPLC chromatograms of the bioconjugates......S3

Analytical data

Summarized analytical data of the daunomycin-tuftsin bioconjugates

Table S1. Characteristics of daunomycin-tuftsin conjugates

Number	Conjugates	R_t (min)[a]	$MW_{calc.}$ / $MW_{meas.}$[b]
**1**	H-TK(Dau=Aoa)PR-OH	19.8	1082.5/1082.4
**2**	For-TK(Dau=Aoa)PR-OH	20.4	1110.5/1110.4
**3**	H-TK(Dau=Aoa-GFLG)PR-OH	21.8	1456.7/1456.7
**4**	H-[TK(Dau=Aoa)PR]$_2$-OH	19.1	2147.0/2147.1
**5**	For-[TK(Dau=Aoa)PR]$_2$-OH	19.5	2175.0/2175.1
**6**	H-[TK(Dau=Aoa-GFLG)PR]$_2$-OH	22.2	2895.4/2895.3

[a] Knauer RP-HPLC; Nucleosil C18 column (5 μm, 100 Å; 250×4.6 mm) gradient: 0 min 2% B, 5 min 2% B, 30 min 90% B; eluents: 0.1% TFA in water (A) and 0.1% TFA in acetonitrile-water 80:20%, v/v (B); flow rate: 1 mL/min, detection: λ = 220 nm.

[b] Bruker Daltonics Esquire 3000+ ESI-MS, 10 μL/min flow rate, positive ion mode in the *m/z* 50–2000 range.

Mass spectra and analytical RP-HPLC chromatograms of the bioconjugates

H-TK(Dau=Aoa)PR-OH (**1**)

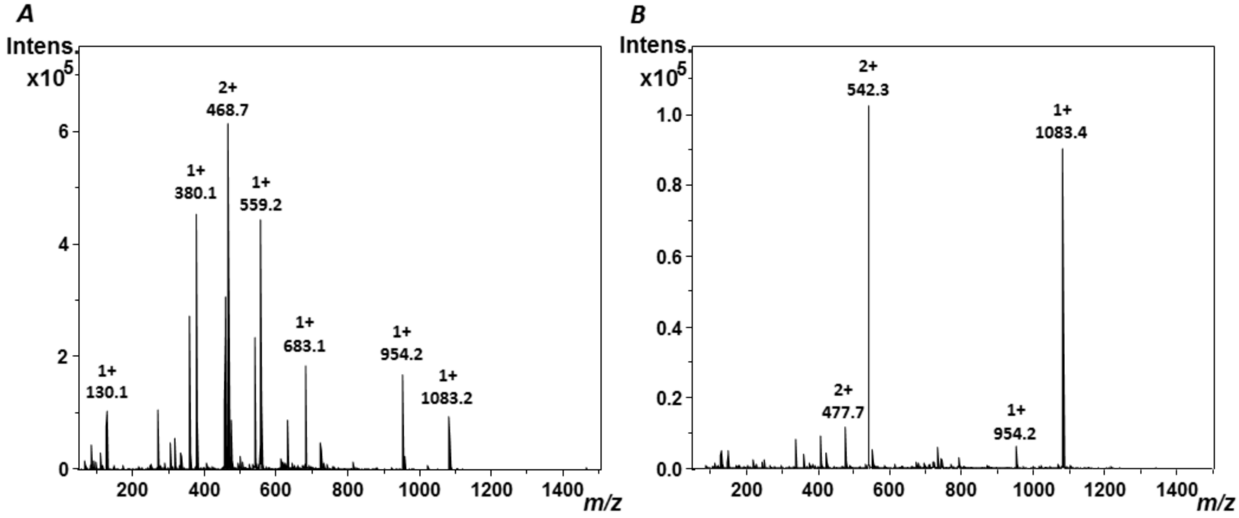

Figure S1. ESI-MS spectra of **1** under the commonly used ESI-MS conditions (acetonitrile-water (50:50%, v/v), 0.1% acetic acid; cap. exit: 136V; *A*) and under the optimized conditions (NH_4OAc buffer (50 mM, pH = 6.7) and acetonitrile (50:50%, v/v); cap. exit: 5V; *B*)

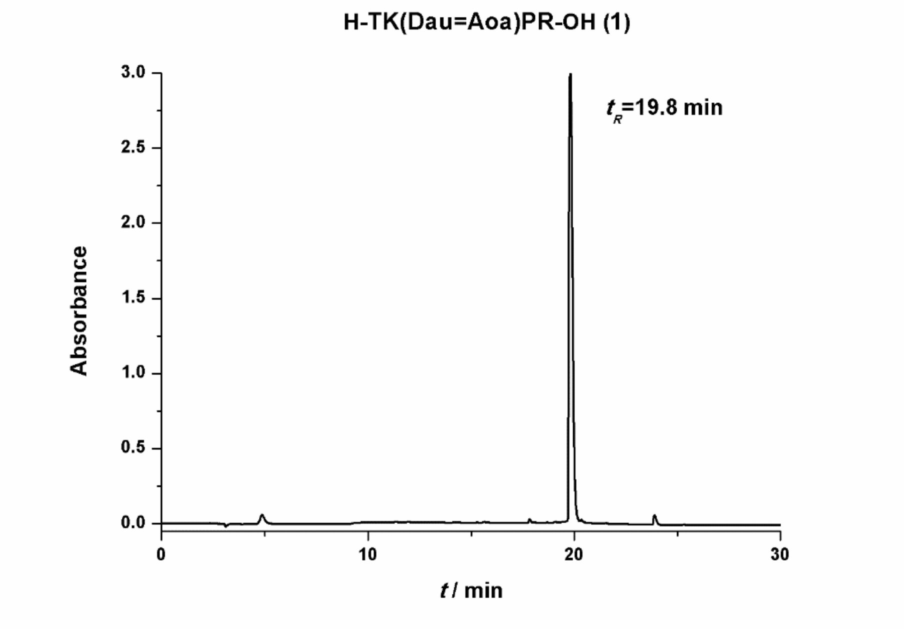

Figure S2. Analytical chromatogram of **1**
(Knauer RP-HPLC; Nucleosil C18 column (5 μm, 100 Å; 250×4.6 mm) gradient: 0 min 2% B, 5 min 2% B, 30 min 90% B; eluents: 0.1% TFA in water (A) and 0.1% TFA in acetonitrile-water 80:20%, v/v (B); flow rate: 1 mL/min, detection: λ = 220 nm)

For-TK(Dau=Aoa)PR-OH (**2**)

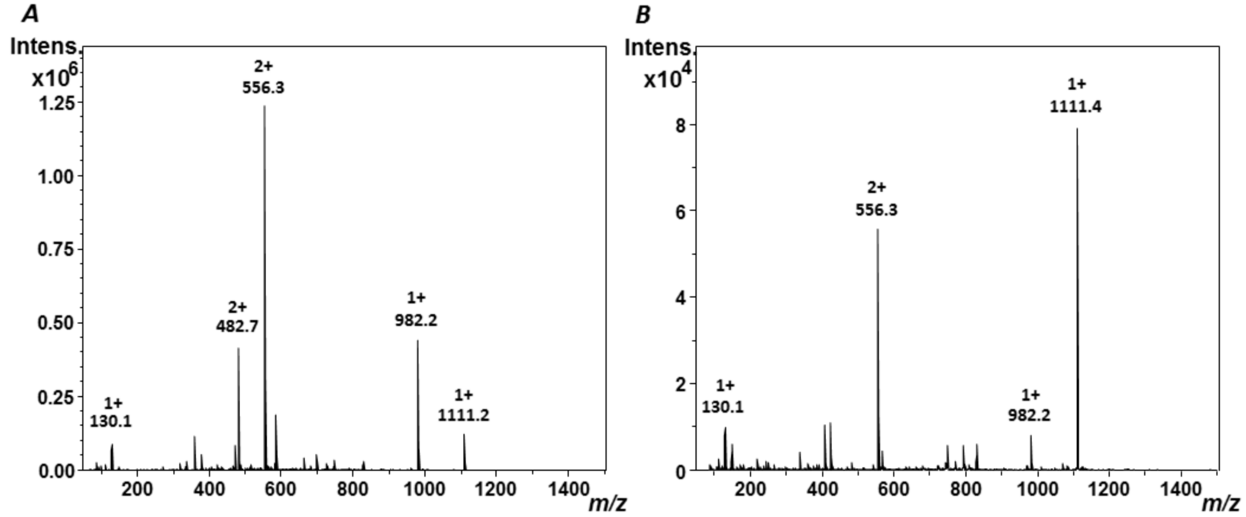

Figure S3. ESI-MS spectra of **2** under the commonly used ESI-MS conditions (acetonitrile-water (50:50%, v/v), 0.1% acetic acid; cap. exit: 136V; *A*) and under the optimized conditions (NH_4OAc buffer (50 mM, pH = 6.7) and acetonitrile (50:50%, v/v); cap. exit: 5V; *B*)

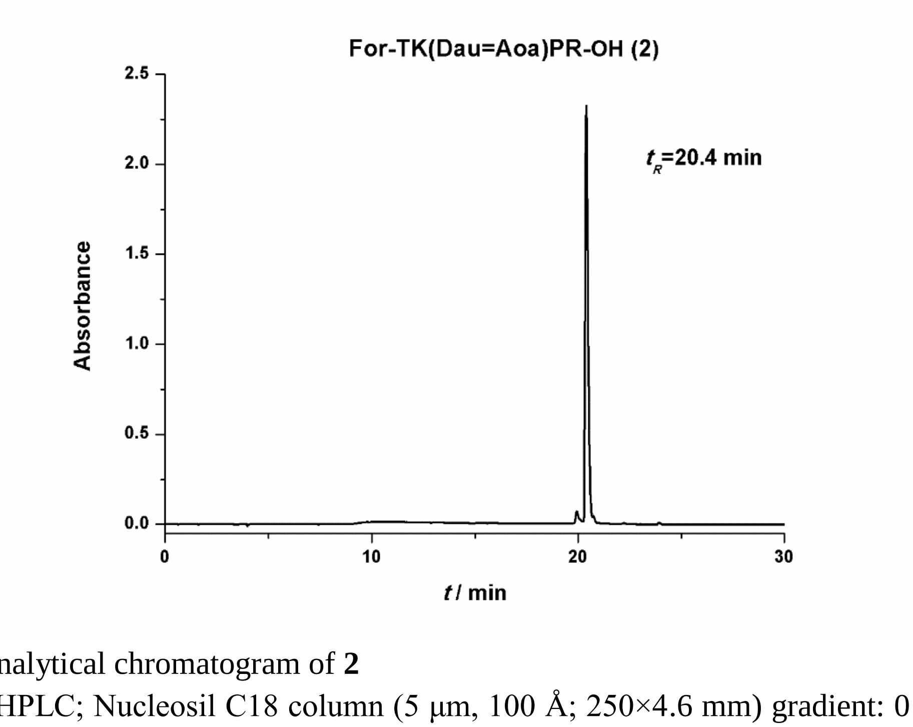

Figure S4. Analytical chromatogram of **2**
(Knauer RP-HPLC; Nucleosil C18 column (5 µm, 100 Å; 250×4.6 mm) gradient: 0 min 2% B, 5 min 2% B, 30 min 90% B; eluents: 0.1% TFA in water (A) and 0.1% TFA in acetonitrile-water 80:20%, v/v (B); flow rate: 1 mL/min, detection: λ = 220 nm)

H-TK(Dau=Aoa-GFLG)PR-OH (**3**)

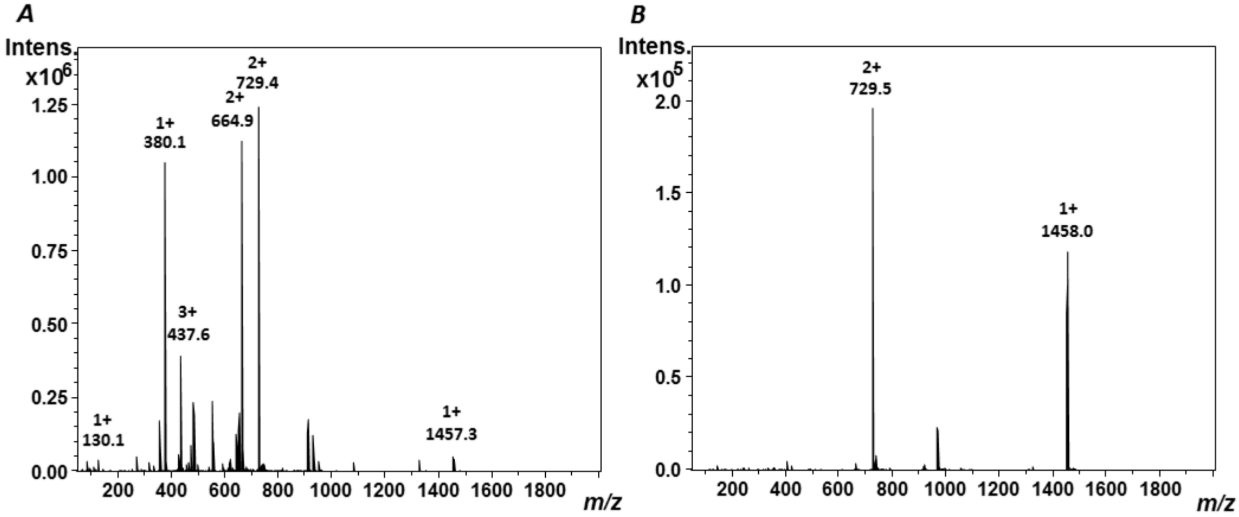

Figure S5. ESI-MS spectra of **3** under the commonly used ESI-MS conditions (acetonitrile-water (50:50%, v/v), 0.1% acetic acid; cap. exit: 143V; *A*) and under the optimized conditions (NH_4OAc buffer (50 mM, pH = 6.7) and acetonitrile (50:50%, v/v); cap. exit: 5V; *B*)

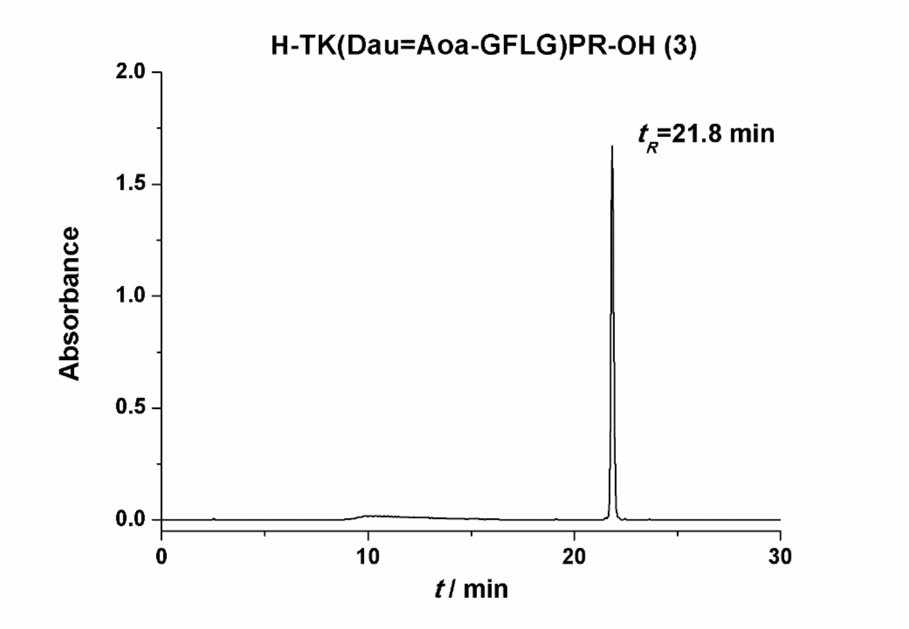

Figure S6. Analytical chromatogram of **3**
(Knauer RP-HPLC; Nucleosil C18 column (5 µm, 100 Å; 250×4.6 mm) gradient: 0 min 2% B, 5 min 2% B, 30 min 90% B; eluents: 0.1% TFA in water (A) and 0.1% TFA in acetonitrile-water 80:20%, v/v (B); flow rate: 1 mL/min, detection: λ = 220 nm)

H-TK(Dau=Aoa-GFLG)PR-OH sugar-lost (**3**)

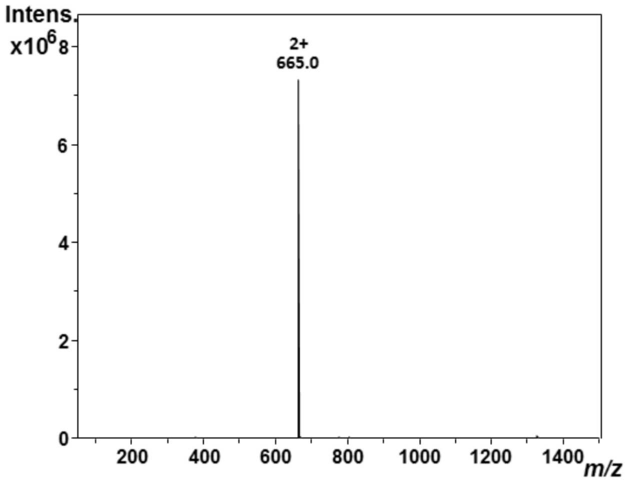

Figure S7. ESI-MS spectrum of sugar-lost, purified **3** under the commonly used ESI-MS conditions (acetonitrile-water (50:50%, v/v), 0.1% acetic acid; cap. exit: 143V)

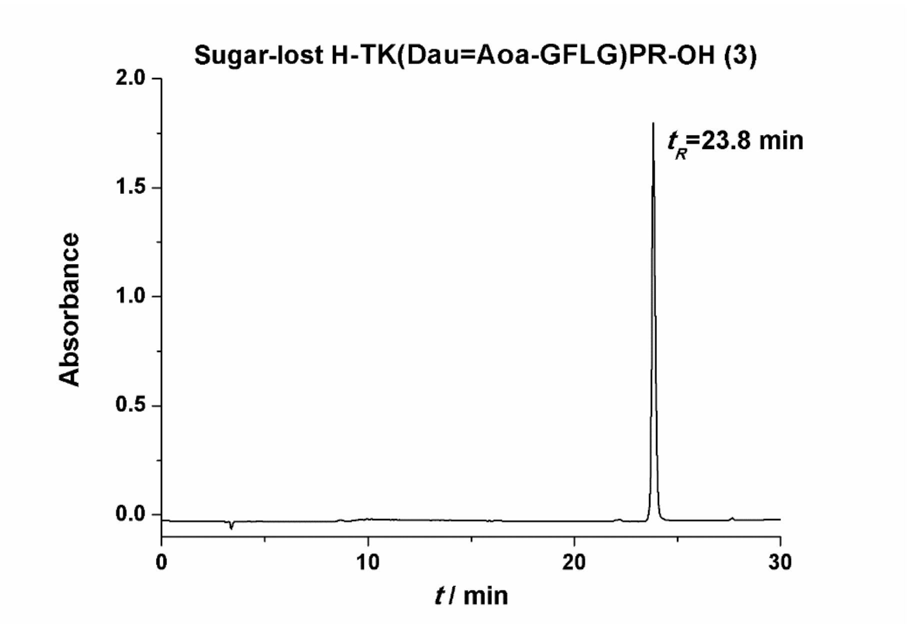

Figure S8. Analytical chromatogram of sugar-lost **3**
(Knauer RP-HPLC; Nucleosil C18 column (5 µm, 100 Å; 250×4.6 mm) gradient: 0 min 2% B, 5 min 2% B, 30 min 90% B; eluents: 0.1% TFA in water (A) and 0.1% TFA in acetonitrile-water 80:20%, v/v (B); flow rate: 1 mL/min, detection: λ = 220 nm)

H-[TK(Dau=Aoa)PR]$_2$-OH (**4**)

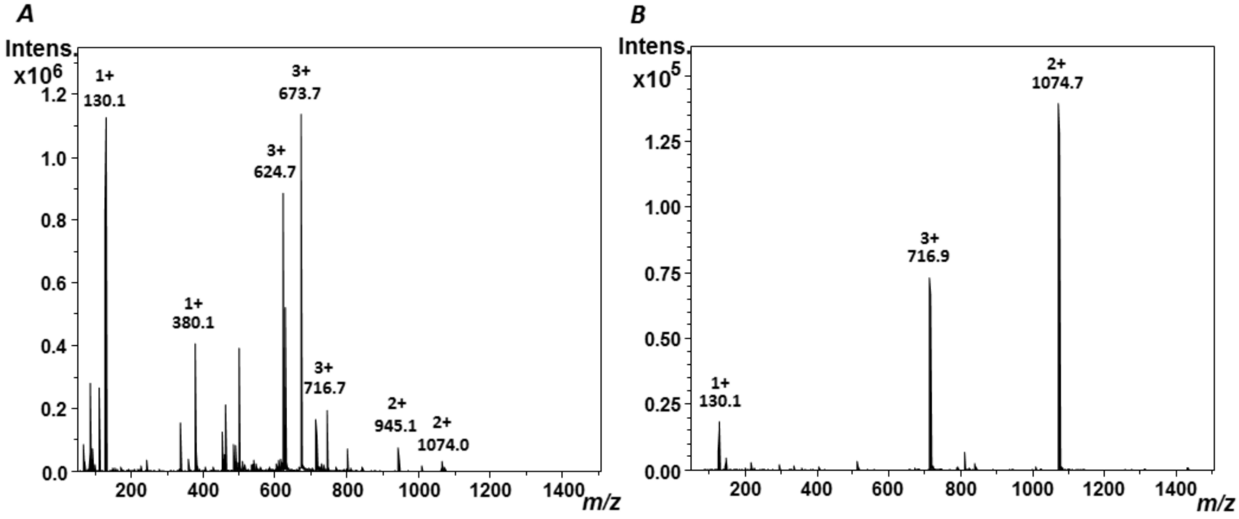

Figure S9. ESI-MS spectra of **4** under the commonly used ESI-MS conditions (acetonitrile-water (50:50%, v/v), 0.1% acetic acid; cap. exit: 143V; *A*) and under the optimized conditions (NH_4OAc buffer (50 mM, pH = 6.7) and acetonitrile (50:50%, v/v); cap. exit: 5V; *B*)

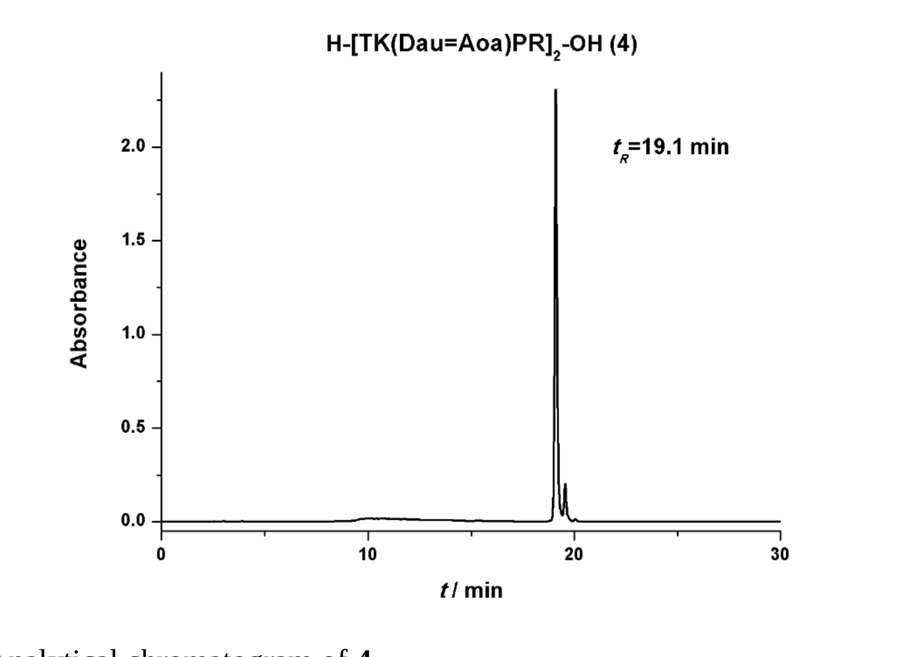

Figure S10. Analytical chromatogram of **4**
(Knauer RP-HPLC; Nucleosil C18 column (5 µm, 100 Å; 250×4.6 mm) gradient: 0 min 2% B, 5 min 2% B, 30 min 90% B; eluents: 0.1% TFA in water (A) and 0.1% TFA in acetonitrile-water 80:20%, v/v (B); flow rate: 1 mL/min, detection: λ = 220 nm)

For-[TK(Dau=Aoa)PR]$_2$-OH (**5**)

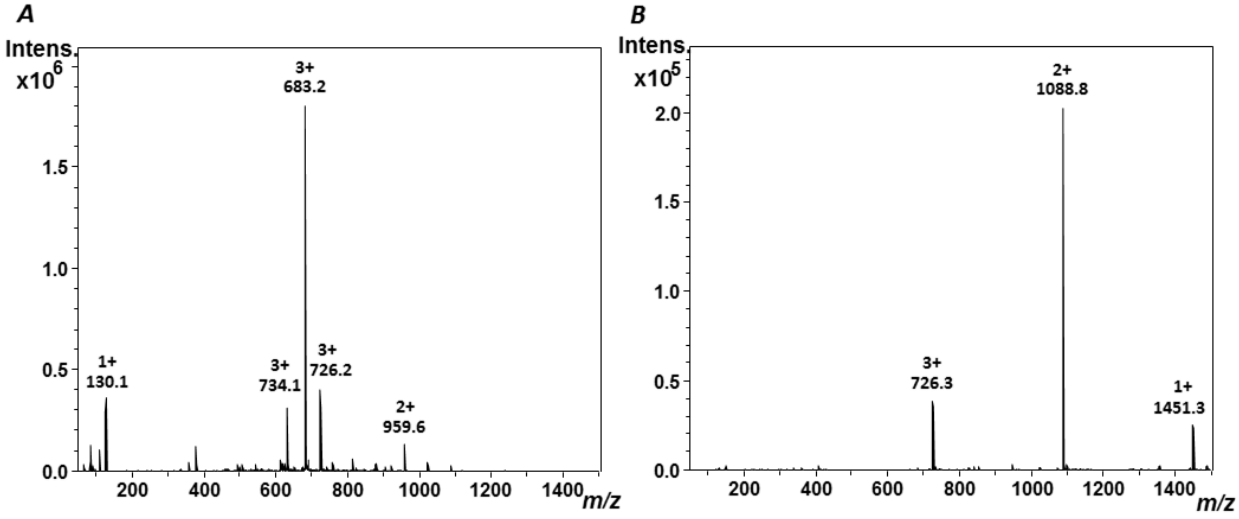

Figure S11. ESI-MS spectra of **5** under the commonly used ESI-MS conditions (acetonitrile-water (50:50%, v/v), 0.1% acetic acid; cap. exit: 143V; *A*) and under the optimized conditions (NH_4OAc buffer (50 mM, pH = 6.7) and acetonitrile (50:50%, v/v); cap. exit: 5V; *B*)

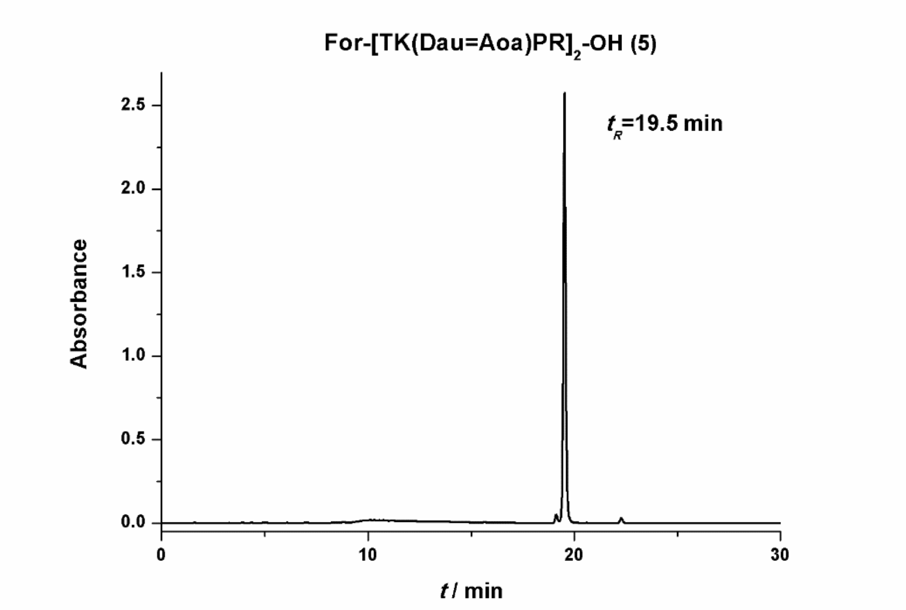

Figure S12. Analytical chromatogram of **5**
(Knauer RP-HPLC; Nucleosil C18 column (5 µm, 100 Å; 250×4.6 mm) gradient: 0 min 2% B, 5 min 2% B, 30 min 90% B; eluents: 0.1% TFA in water (A) and 0.1% TFA in acetonitrile-water 80:20%, v/v (B); flow rate: 1 mL/min, detection: λ = 220 nm)

H-[TK(Dau=Aoa-GLFG)PR]$_2$-OH (**6**)

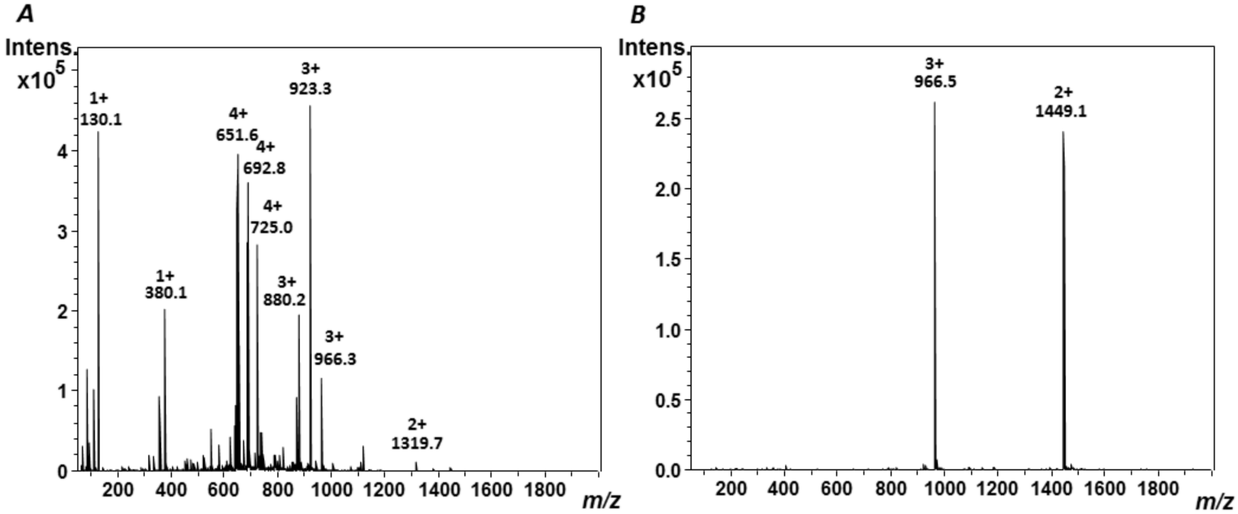

Figure S13. ESI-MS spectra of **6** under the commonly used ESI-MS conditions (acetonitrile-water (50:50%, v/v), 0.1% acetic acid; cap. exit: 143V; *A*) and under the optimized conditions (NH_4OAc buffer (50 mM, pH = 6.7) and acetonitrile (50:50%, v/v); cap. exit: 5V; *B*)

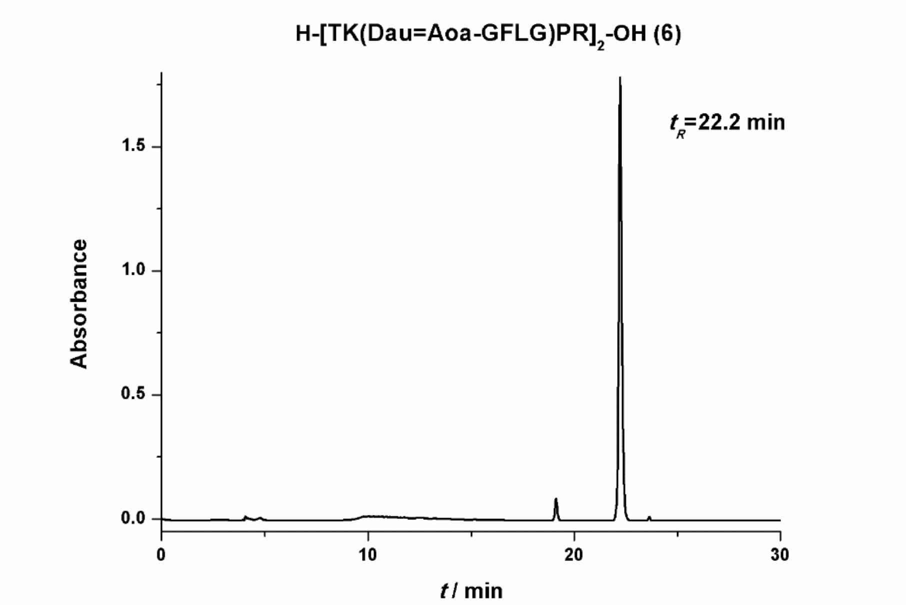

Figure S14. Analytical chromatogram of **6**
(Knauer RP-HPLC; Nucleosil C18 column (5 µm, 100 Å; 250×4.6 mm) gradient: 0 min 2% B, 5 min 2% B, 30 min 90% B; eluents: 0.1% TFA in water (A) and 0.1% TFA in acetonitrile-water 80:20%, v/v (B); flow rate: 1 mL/min, detection: λ = 220 nm)
